# Reconstructing the Future: Long-Term Outcome of a Case of Scaphoid Dislocation With Concurrent Hamate and Triquetrum Fractures

**DOI:** 10.7759/cureus.70449

**Published:** 2024-09-29

**Authors:** Suvank Rout, Hyder Tahir, Al Julanda Al Maskari, Sreelakshmi Suresh

**Affiliations:** 1 Trauma and Orthopaedics, Colchester General Hospital, Colchester, GBR; 2 Medicine, Topiwala National Medical College & Bai Yamunabai Laxman (BYL) Nair Charitable Hospital, Mumbai, IND; 3 General Surgery, Colchester General Hospital, Colchester , GBR

**Keywords:** hamate, trauma, wrist, dislocation, scaphoid

## Abstract

Scaphoid dislocations are an extremely rare injury. The authors herein report a 60-year-old male who was managed with open reduction and internal fixation with Kirschner wires (K-wires) and scapholunate ligament stabilisation. The aim of this case report is to comprehensively present this unusual injury along with its treatment and long-term follow-up outcome alongside a literature review to aid surgeons confronted with this rare pathology.

## Introduction

Scaphoid dislocations are an extremely rare injury, and case reports are available in the English literature. These usually present a challenge to orthopaedic surgeons given the limited experience and published reports of these potentially life-changing nature of the injuries. The first isolated scaphoid dislocations were reported by Ely in 1903 of a patient due to a likely crush injury in a road traffic collision (RTC) [1]. The majority of the case reports since have been due to RTCs [2-3]. These injuries and their mechanisms have been documented by Lueng et al. [4] in 1998. 

This form of injury is rare due to the complex anatomy and the multiplanar stability that is provided by the various carpal ligaments. The most common forces involved in the occurrence of this injury in literature have been those of power grip, dorsiflexion, and ulnar deviation [5-6]. 

Since the first description of the carpal scaphoid dislocation, the historical treatment of choice has been closed reduction. This has often led to limitations of movements or the thumb. Kirschner-wire (K-wire) fixation and simultaneous ligament injury repair have now become the current treatment of choice in the last two decades. The most common complications of note from surgical treatment have been those of residual subluxation [7]. Avascular necrosis and median nerve compromise have also been reported [7-8].

We present a case of a scaphoid dislocation with associated hamate and triquetrum fractures and its management and long-term follow-up, to add to the limited literature of these complex carpal injuries. 

## Case presentation

**Figure 1:**
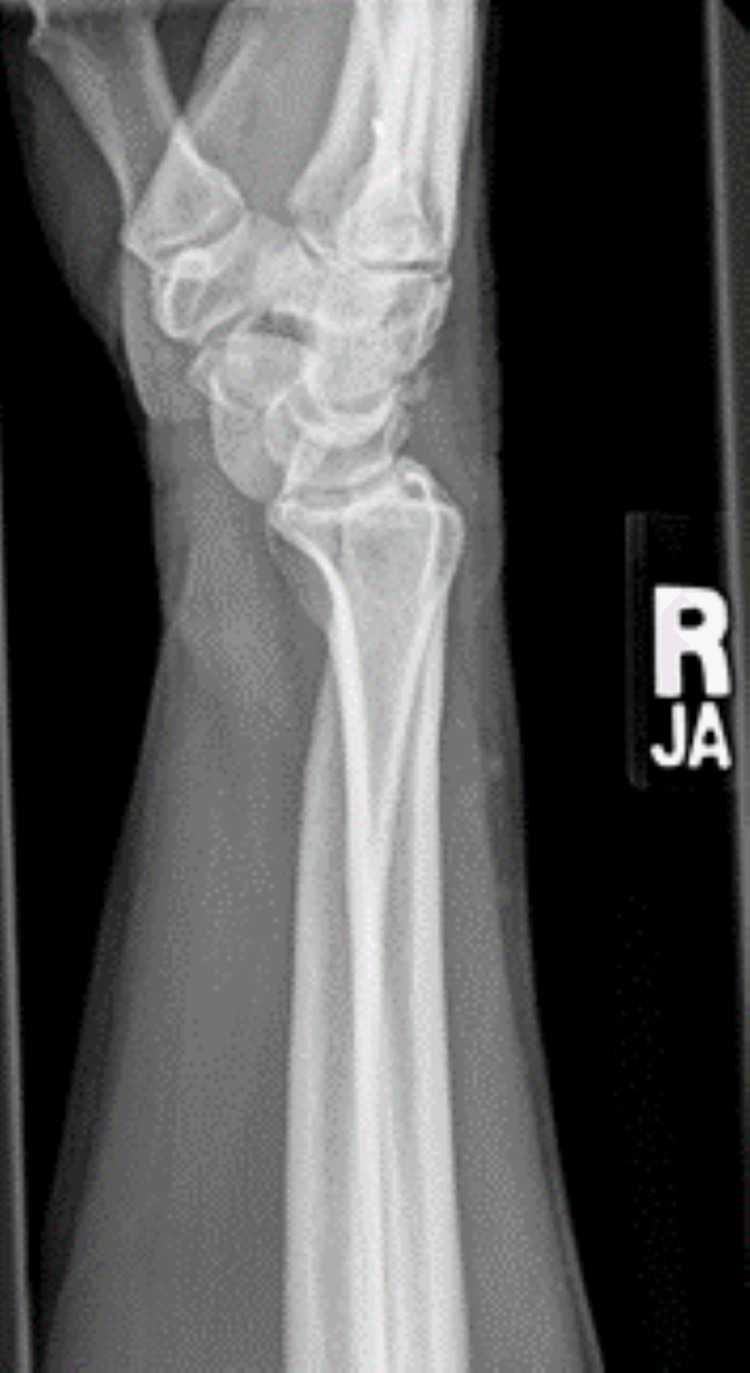
Lateral view of the radiograph shows acute radiopalmar dislocation of the carpal scaphoid

**Figure 2:**
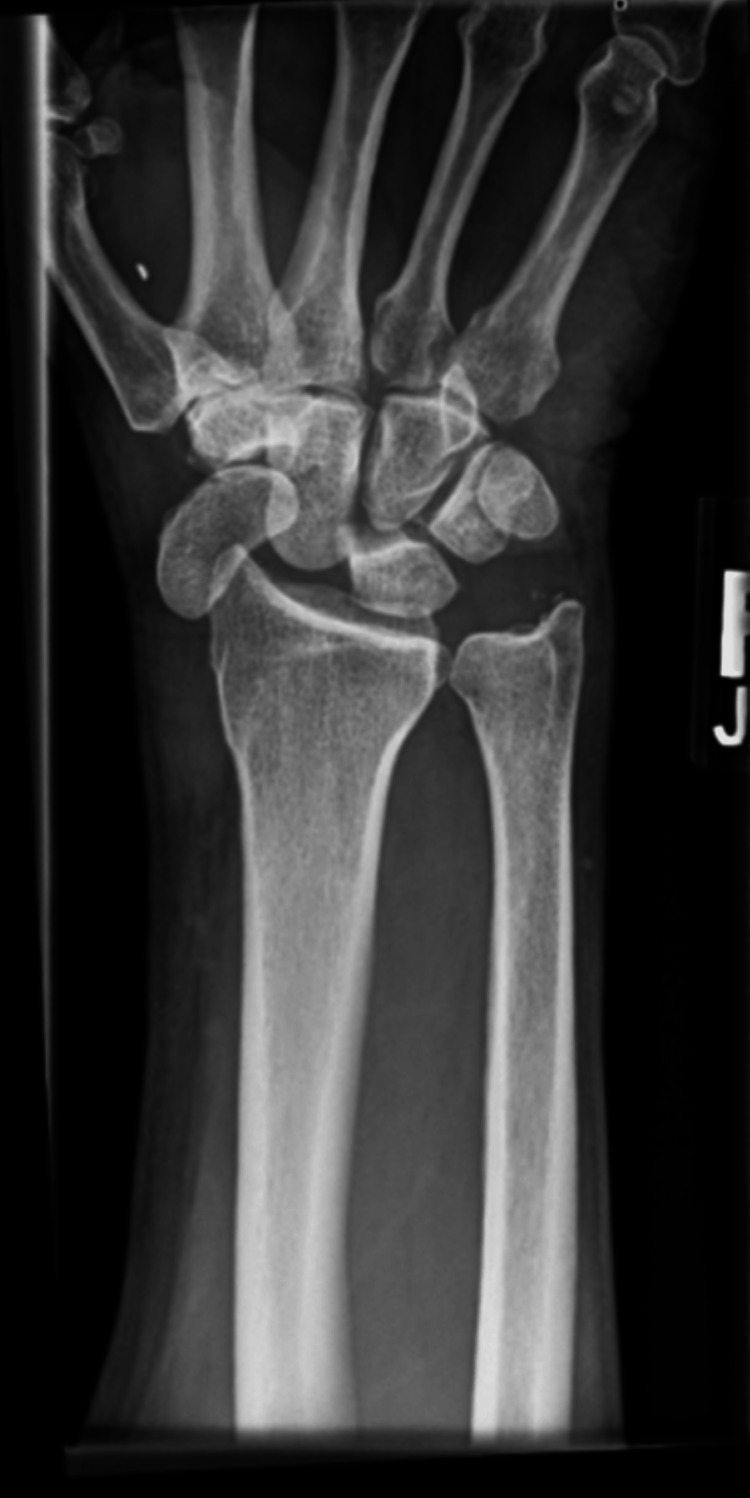
Anteroposterior view of the radiograph shows acute radiopalmar dislocation of the carpal scaphoid, with a metal foreign body in the first webbed space (complex fracture dislocation of intercarpal, radiocarpal, and carpometacarpal joints)

**Figure 3:**
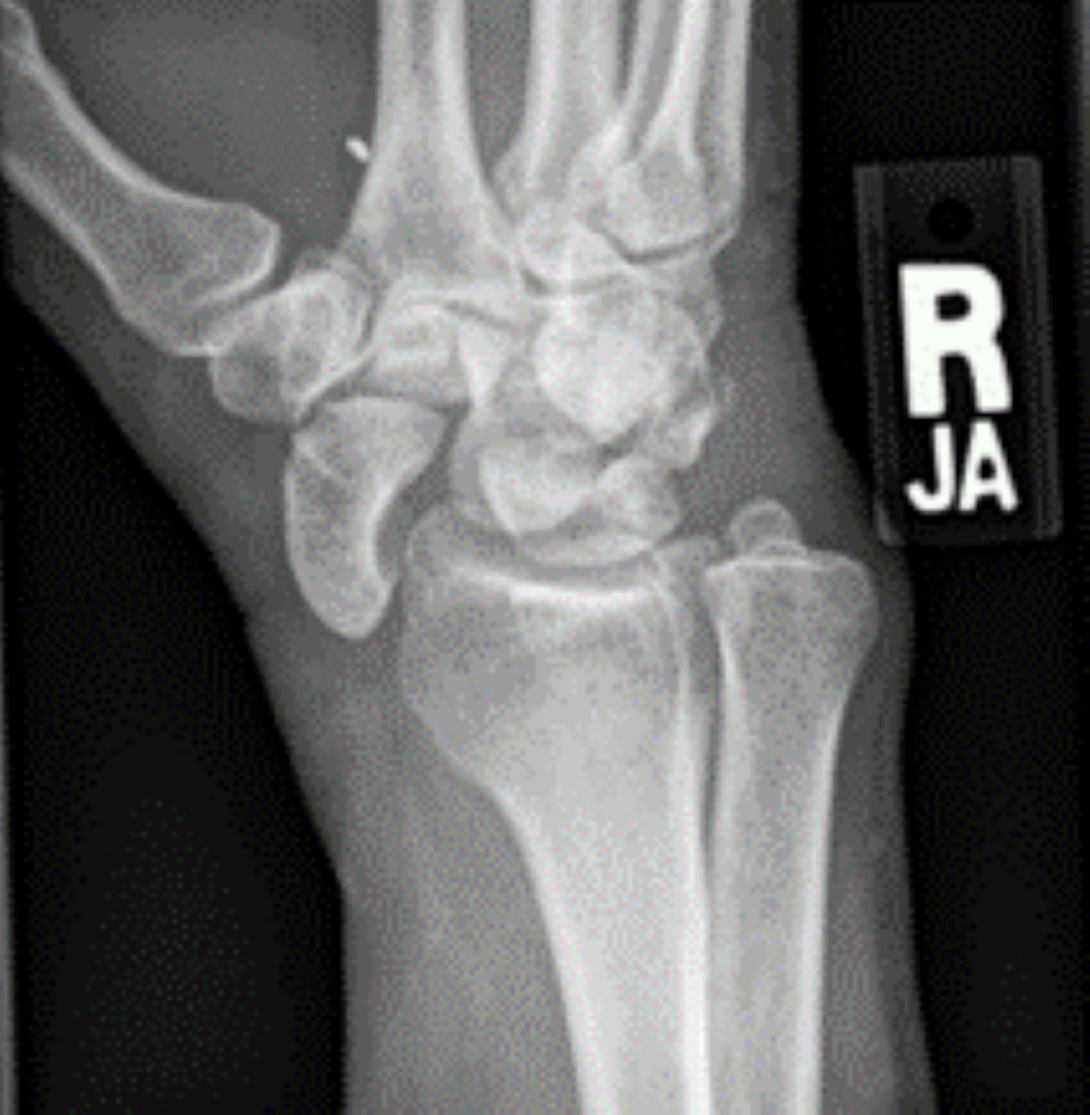
Scaphoid view of the radiograph shows the carpo-radial dislocation of the scaphoid along with a metal foreign body in the first webbed space

**Figure 4:**
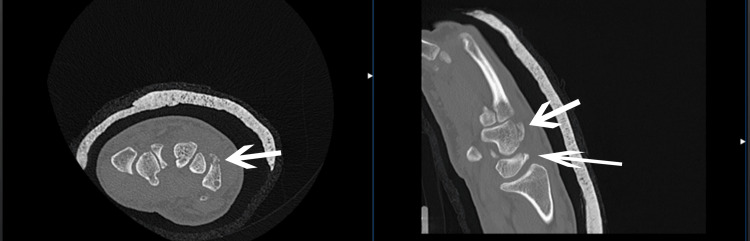
A CT scan demonstrates dorsal fractures of the hamate and triquetral bones (arrows)

A 60-year-old, left-hand dominant male presented to our institution after sustaining a closed injury to his right hand caused by a high-energy entrapment of his wrist around the steering wheel of his van whilst involved in a 60-mph van vs. heavy goods vehicle RTC, in which his van spun and hit the central reservation. The patient presented with complaints of severe pain and loss of active and passive right wrist flexion. On examination, there was pain, swelling, and a bony prominence volar to the radial styloid process. There was no neurovascular deficit. Lateral (Figure 1), anteroposterior (Figure 2), and scaphoid view (Figure 3) radiographs showed that the patient had a volar dislocation of the scaphoid and a foreign body in the palmar aspect of the hand. A computed tomography (CT) scan was then performed (Figure 4), which demonstrated a volar dislocation of the scaphoid and fractures in the dorsal aspect of the hamate and triquetral bones.

Management

The patient was initially placed in a plaster of Paris (PoP) dorsal back slab for his right wrist in the same position without any attempt at manipulation in the emergency department. The patient then underwent manipulation under anaesthesia with the aim of closed reduction with 10 mg intravenous morphine in the operating theatre within 24 hours. In-line traction via finger traps with 5 kg weights was attempted but did not result in the reduction of the dislocation. Screened manual traction with ulnar deviation of the carpus under fluoroscopic guidance and manual reduction of the carpal scaphoid proximal pole resulted in a good reduction of the scaphoid bone. Post-reduction examination revealed gross laxity of the scapholunate ligament. 

**Figure 5:**
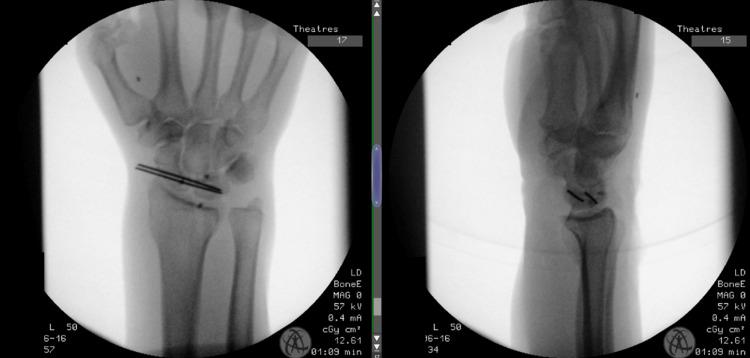
Intraoperative anteroposterior (left) and lateral (right) fluoroscopic images following reduction, fixation with K-wires, and anchors used for scapholunate ligament repair are visible. K-wires: Kirschner wires

The patient thus underwent open reduction through the dorsal approach of the scaphoid. The dorsal wrist capsule was partially disrupted, which was surgically converted into an inverted T-capsulotomy. The dorsal remnants of the scapholunate ligament were identified and released from the capsule. There was complete avulsion from the proximal pole of the scaphoid and partial avulsion from the lunate insertion. Joystick 1.1 mm K-wires were inserted into the scaphoid and lunate to reduce the scapholunate joint and restore the scapholunate relationship. Two 1.1 mm K-wires were used to transfix the scaphoid to the lunate. A 2.2 mm suture anchor was inserted into the scaphoid at the site of the avulsed scapholunate ligament insertion. A second 2.2 mm anchor was placed through the avulsion footprint in the lunate. The scapholunate ligament origin and bony fragment were sutured to the dorsal lunate. The trans-scaphoid wires were cut below the level of the skin. A third anchor was used to repair the capsulotomy via a dorsal rim 2.2 mm anchor. Final intraoperative fluoroscopy images are demonstrated in Figure 5.

Postoperative course

**Figure 6:**
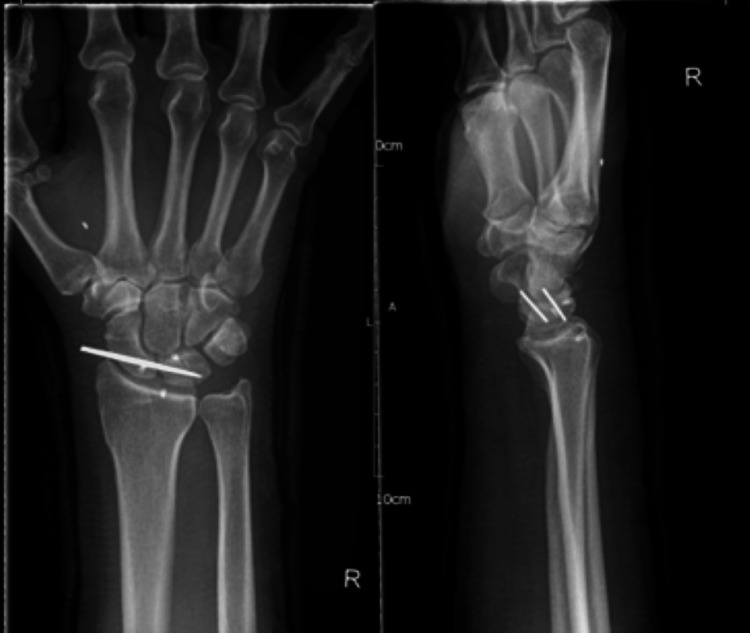
Anteroposterior (left) and lateral (right) radiographs of the right wrist made four weeks after open reduction and internal fixation with K-wires, and anchors used for scapholunate ligament repair visible. K-wires: Kirschner wires

Postoperatively, a below-elbow PoP in a neutral wrist position was applied for two weeks. This was then changed to a below-elbow synthetic cast, which was in place for a period of four to six weeks, after which K-wire removal was planned. The patient was discharged the same day and commenced hand therapy within two weeks of surgery, as he complained of postoperative hand weakness to restore motion and strength. Plain radiographs demonstrate appearances four weeks postoperatively (Figure 6).

**Figure 7:**
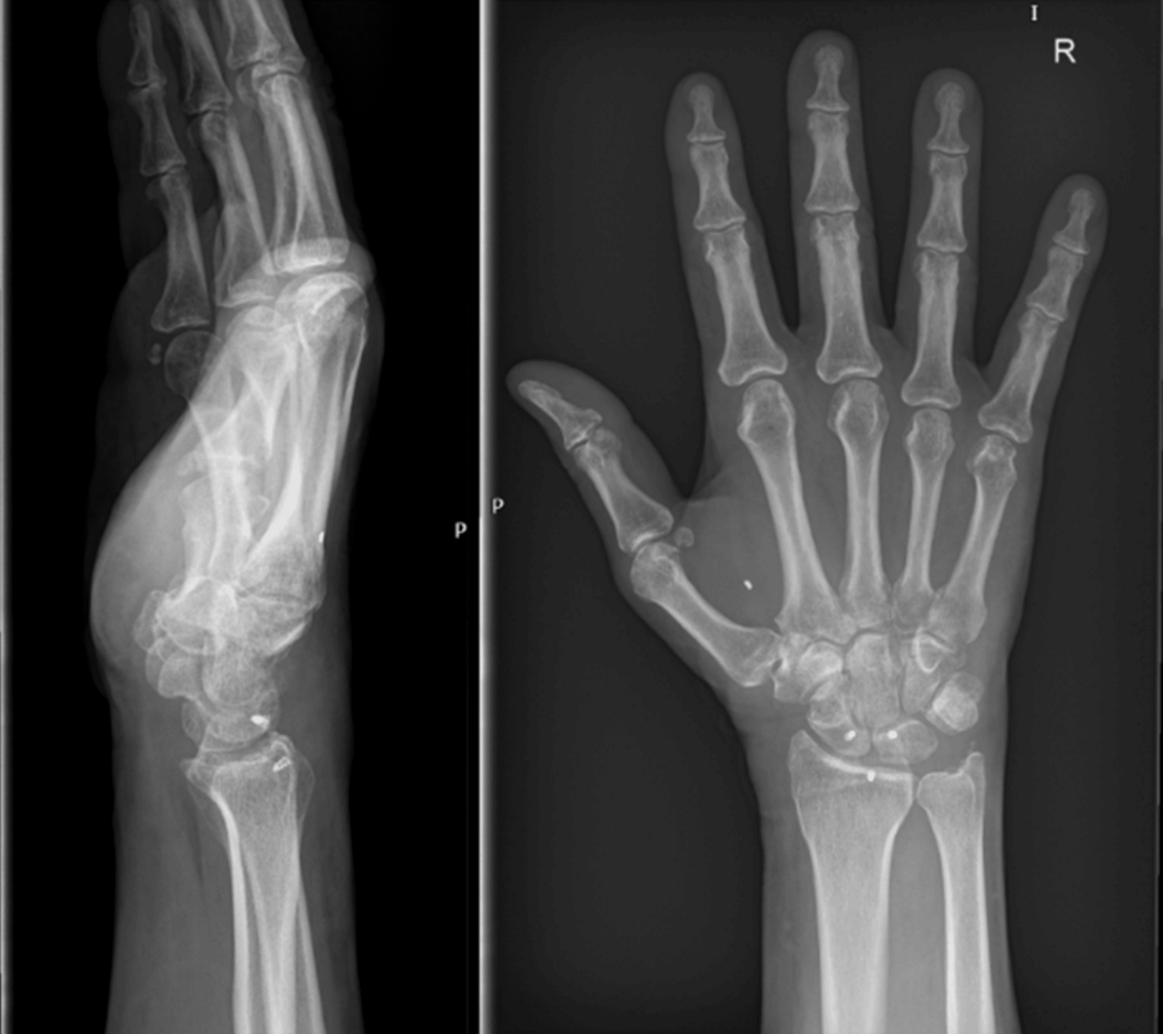
Anteroposterior and lateral views of the radiographs of the right wrist were made six weeks after the removal of K-wires. Anchors used for scapholunate ligament repair remain in situ. K-wire: Kirschner wire

The K-wires were removed eight weeks postoperatively. The patient was reviewed six weeks after the removal of the K-wires from his right wrist following scapholunate ligament reconstruction. The patient was happy with the outcome he had achieved. He had no complaints of pain and had good grip strength in his hand. He did mention slight stiffness compared to the contralateral wrist. On clinical examination, he had a range of motion of 20° extension to 40° flexion. He was advised to continue physiotherapy for his hand. Plain radiographs demonstrated no evidence of carpal subluxation, avascular necrosis, or degenerative arthritis (Figure 7).

**Figure 8:**
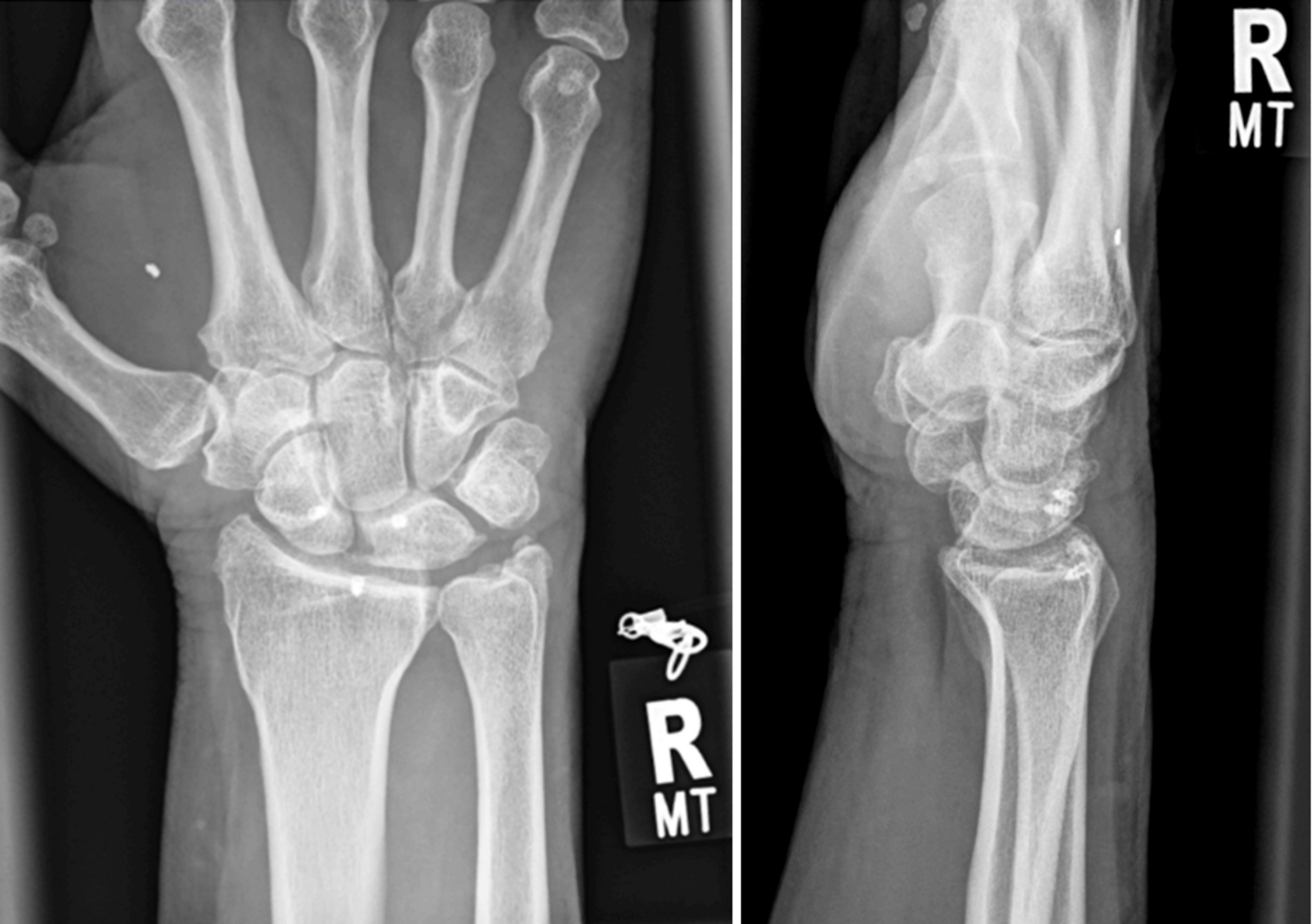
Anteroposterior (left) and lateral (right) radiographs 24 months postoperatively show no scapholunate subluxation or evidence of avascular necrosis. Anchors used for scapholunate ligament repair remain in situ.

**Figure 9:**
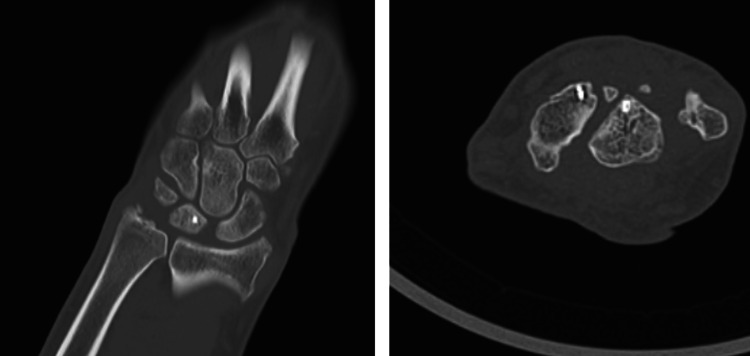
Coronal (left) and axial (right) sections of the CT scan 24 months postoperatively demonstrate no scapholunate dislocation or avascular necrosis. Anchors used for scapholunate ligament repair remain in situ. The hamate and triquetrum fractures have united in a satisfactory position.

**Figure 10:**
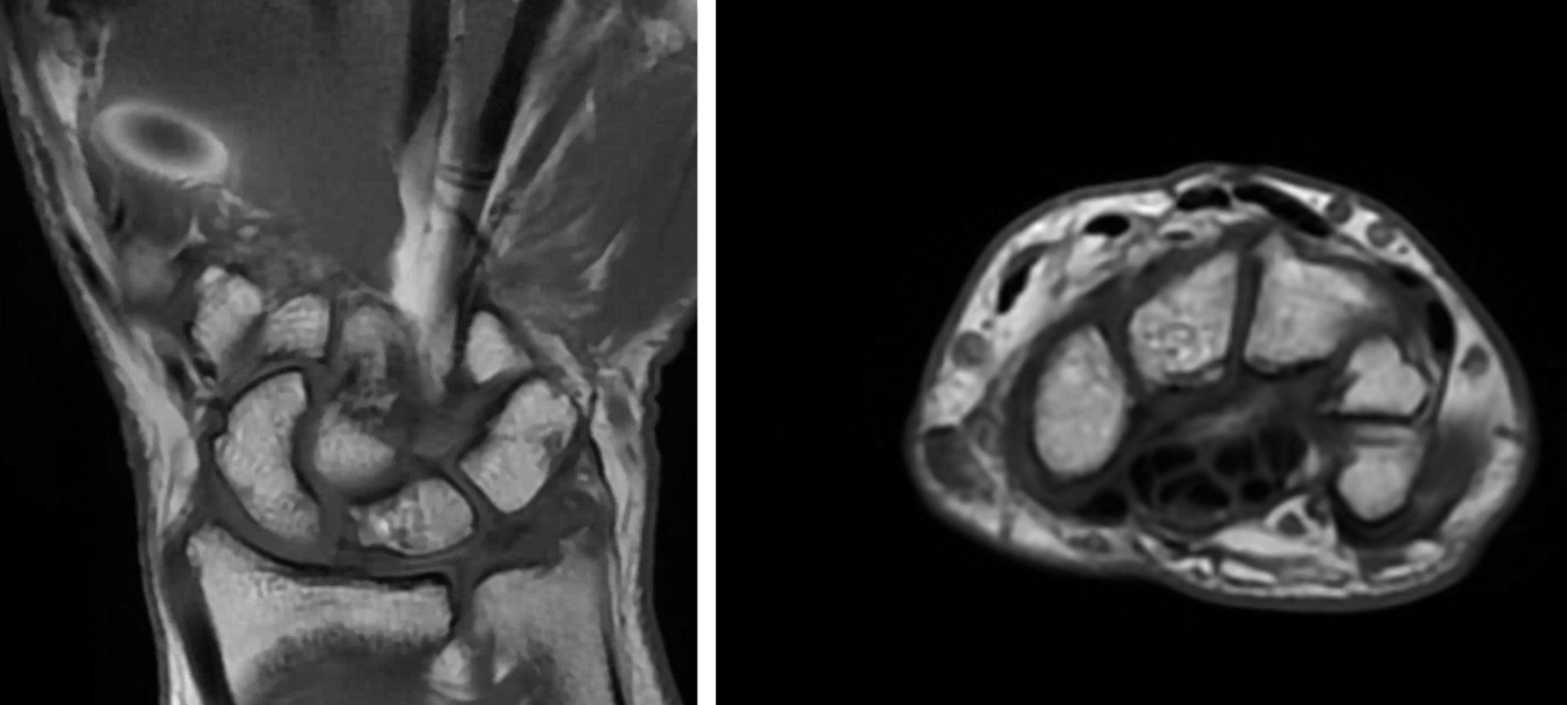
MRI scans with coronary T1 (left) and axial T1 (right) views 24 months postoperatively show no carpal subluxation nor avascular necrosis. There is no signal change in the carpal ligaments.

The patient was followed up after a period of 24 months, where the patient underwent repeat plain radiographs (Figure 8), CT scans (Figure 9), and MRI scans (Figure 10) of the wrist. Plain radiographs did not demonstrate any further complications. The CT scan demonstrated satisfactory carpal alignment as well as the position of anchors used in the ligament reconstruction. The MRI demonstrated no evidence of avascular necrosis, no carpal instability, no widening of the scapholunate joint, and an intact scapholunate ligament.

The patient stated that there was restoration of grip strength, and clinically it was noted that he had an active range of motion from 60° in dorsiflexion to 60° in palmar flexion and full pronation and supination of the wrist. On clinical examination, he had good ulnar and radial deviation. The patient stated that though he has some discomfort in his wrist, he was satisfied with the result. 

## Discussion

Whilst isolated scaphoid dislocations have been reported, their presentation with concurrent fractures of other carpal bones in the same limb is exceptional. The authors encountered such a case in the emergency department, and the necessity for optimal patient care led them to carefully review the available literature. This effort was particularly demanding since the literature reports individual cases and frequently relies solely on the surgeon’s experience. 

In this case report, we presented a rare instance of scaphoid dislocation accompanied by fractures of the hamate and triquetrum, highlighting the complexities in the management of such injuries. The scaphotrapezial, scaphocapitate, and scapholunate interroseous ligaments contribute to the scapholunate stability and are often injured in scaphoid dislocation [9]. It is hypothesised that the pattern of failure of ligaments in the dislocation of scaphoid could be radioscaphocapitate, scapholunate interroseous, long radiolunate, and scaphotrapezial ligaments [5]. The most common forces involved in the occurrence of this injury in literature have been those of power grip, dorsiflexion, and ulnar deviation [4-7]. An additional axial force from distal to proximal through the metacarpals may also cause disruption of the carpus. 

The rarity of this combination underscores the importance of thorough clinical evaluation and advanced imaging techniques to accurately assess the extent of injury. Initial diagnostic evaluation must include standard anteroposterior, lateral, oblique, and radio-ulnar deviation radiographs. A CT scan will help in assessing associated bony injury as well as provide 3D visualisation for the operating surgeon, as was noted in this case presentation.

Treatment modalities then shifted towards percutaneous fixation after closed reduction or open reduction and to open reduction and ligament reconstruction with internal fixation. There is nearly universal loss of range of wrist motion in early case reports [4]. In cases where arthritic changes in a wrist are present due to carpal instability, the best results were obtained with partial wrist arthrodesis. 

Our patient underwent prompt surgical intervention and treatment the day after the injury, which was followed by cast immobilisation for a total of eight weeks, which was followed by the timely commencement of specialised rehabilitation and hand therapy after cast removal. Acute scaphoid dislocation has historically been treated with closed reduction and casting. Fortunately, the patient in our case demonstrated a favourable postoperative course with significant improvement in pain, range of motion, and grip strength. A successful outcome can be attributed to prompt diagnosis, meticulous surgical technique, and appropriate postoperative rehabilitation. 

A review of the existing literature revealed two cases involving scaphoid dislocation with concomitant fractures of other carpal bones [10-11]. Aditya et al. in 2013 reported the case of a 20-year-old male who sustained a scaphoid dislocation with an associated fracture of the ipsilateral radial styloid and fracture and proximal migration of the distal carpal row [10]. Following attempt at closed reduction, as in our case, their patient underwent open reduction with K-wire fixation of the hamate fracture and reconstruction of the damaged scapholunate ligament with flexor carpi radialis tendon graft. Postoperatively, the patient had an external fixator frame applied. Both K-wire and external fixators were removed at six weeks. They report comparable functional outcomes to ourselves at 26 months with no evidence of avascular necrosis. Our case report adds to the evidence that demonstrates open reduction and ligament reconstruction can confer good long-term outcomes and that application of an external fixation frame is optional for the operating surgeon.

The second case, reported by Liu et al. in 2022, involved a 59-year-old male who sustained a scaphoid dislocation with associated 'chip' fractures of the capitate and hamate [11]. Whilst the morphology of the concomitant carpal fractures was similar to that found in our case, their management included arthroscopic-assisted reduction, again after a failed attempt at closed reduction, with K-wire fixation of the position of the scaphoid to capitate and lunate. The extent of follow-up in this case was six months, whereupon the patient demonstrated a range of motion that just mildly decreased compared to the uninjured wrist. There was no evidence of avascular necrosis or residual subluxation.

The scarcity of reported cases underscores the rarity of this injury pattern and the need for continued documentation and sharing of experiences to inform best practices in diagnosis and management. Our case report adds to the evidence that demonstrates open reduction, K-wire fixation, and ligament reconstruction can confer good long-term outcomes.

## Conclusions

In conclusion, a scaphoid dislocation is a rare and challenging clinical entity. This case report contributes to the growing body of evidence regarding the diagnosis and management of such injuries. Through a combination of advanced imaging, surgical intervention, and comprehensive early hand rehabilitation, a positive outcome has been achieved in our patient. This case underscores the importance of continued research and collaboration to enhance our understanding and management of complex carpal injuries.
